# Supplementary material for: Long-term persistence of viral RNA and inflammation in the CNS of macaques exposed to aerosolized Venezuelan equine encephalitis virus
Source: PLoS Pathog. 2022 Jun 13;18(6):e1009946. doi: 10.1371/journal.ppat.1009946 (PMC9232170; doi:10.1371/journal.ppat.1009946)
Supplement: S1 Table — a Virus = isolate used; Age: years; Weight: kg; Dose: log10 pfu. b Rmax = maximum residual difference between actual and predicted temperature. c Duration: hours. d Fever-hours: sum of significant temperature elevations, divided by four to convert to hours. e Ave Elev: average elevation, fever-hours / duration. f Overall = fever for the entire post-challenge period. g First = fever during the first fever period, 0.5–2 dpi. (DOCX) [file ppat.1009946.s001.docx]

| **Virus^a^** | **Macaque** | **Sex** | **Age** | **Weight** | **Dose** | **Overall^f^** | | | | **First^g^** | | | | **Second^h^** | | | |
| --- | --- | --- | --- | --- | --- | --- | --- | --- | --- | --- | --- | --- | --- | --- | --- | --- | --- |
|  | **ID** |  |  |  |  | **Max∆T^b^** | **Duration^c^** | **Fever-Hours^d^** | **Ave Elev^e^** | **Max∆T** | **Duration** | **Fever-Hours** | **Ave Elev** | **Max∆T** | **Duration** | **Fever-Hours** | **Ave Elev** |
| **INH-9813** | 171-16 | M | 7 | 5 | 6.0 | 3.2 | 230.3 | 298.6 | 1.3 | 2.8 | 32.3 | 50.5 | 1.6 | 3.2 | 114.3 | 189.5 | 1.7 |
|  | 170-16 | M | 6 | 5.2 | 6.3 | 2.8 | 186.0 | 168.3 | 0.9 | 2.8 | 23.0 | 31.3 | 1.4 | 2.1 | 79.5 | 78.3 | 1.0 |
|  | 107-18 | F | 5.5 | 3.54 | 6.5 | 1.7 | 38.8 | 38.0 | 1.0 | 1.7 | 23.0 | 24.0 | 1.0 | 1.7 | 15.8 | 14.0 | 0.9 |
|  | 121-16 | F | 6 | 3.6 | 6.8 | 2.6 | 307.3 | 381.4 | 1.2 | 1.7 | 25.0 | 28.8 | 1.2 | 2.6 | 88.8 | 149.4 | 1.7 |
|  | 165-16 | F | 5 | 3.5 | 6.9 | 2.8 | 130.5 | 195.2 | 1.5 | 2.1 | 30.3 | 41.2 | 1.4 | 2.8 | 100.3 | 154.0 | 1.5 |
|  | 6-19* | M | 2 | 2.6 | 6.9 | 5.8 | 122.3 | 375.1 | 3.1 | 5.1 | 46.3 | 135.0 | 2.9 | 5.9 | 65.8 | 220.1 | 3.3 |
|  | 109-18 | F | 5.5 | 3.74 | 7.0 | 4.2 | 308.0 | 448.1 | 1.5 | 3.0 | 37.5 | 58.1 | 1.5 | 4.2 | 115.5 | 229.8 | 2.0 |
|  | 5-19* | F | 2 | 2.4 | 7.0 | 3.9 | 65.5 | 112.3 | 1.7 | 3.4 | 41.5 | 67.0 | 1.6 | 2.2 | 12.3 | 15.8 | 1.3 |
|  | 164-16 | F | 5 | 4 | 7.1 | 2.7 | 131.8 | 146.5 | 1.1 | 2.0 | 18.3 | 22.8 | 1.2 | 2.7 | 97.0 | 114.6 | 1.2 |
|  | 122-16 | F | 5 | 3.2 | 7.2 | 3.8 | 210.8 | 330.0 | 1.6 | 3.8 | 33.0 | 63.4 | 1.9 | 1.6 | 12.3 | 13.1 | 1.1 |
|  | 115-18 | M | 5.5 | 6.7 | 7.2 | 4.8 | 490.0 | 703.1 | 1.4 | 4.5 | 43.5 | 93.0 | 2.1 | 4.8 | 122.5 | 296.5 | 2.4 |
|  | 112-18 | M | 9 | 7.6 | 7.3 | 2.7 | 453.3 | 477.3 | 1.1 | 2.4 | 31.8 | 42.2 | 1.3 | 2.7 | 97.5 | 138.0 | 1.4 |
|  | 111-18 | M | 6 | 7.2 | 7.5 | 3.6 | 370.5 | 435.7 | 1.2 | 3.1 | 44.5 | 64.8 | 1.5 | 3.6 | 109.0 | 201.0 | 1.8 |
|  | 116-18 | M | 9 | 6.9 | 7.8 | 3.5 | 319.5 | 405.8 | 1.3 | 3.0 | 41.8 | 75.1 | 1.8 | 3.5 | 113.0 | 202.0 | 1.8 |
|  | 53-17 | M | 6 | 5.4 | 8.3 | 5.2 | 197.5 | 454.5 | 2.3 | 4.5 | 46.5 | 127.6 | 2.7 | 5.2 | 88.5 | 233.7 | 2.6 |
|  | 54-17 | M | 5 | 6.4 | 8.4 | 4.7 | 163.5 | 302.2 | 1.8 | 4.7 | 40.3 | 91.9 | 2.3 | 3.9 | 43.3 | 82.7 | 1.9 |
| **TrD** | 161-17 | M | 5 | 7.8 | 8.3 | 4.2 | 221.5 | 342.5 | 1.5 | 4.2 | 45.8 | 91.4 | 2.0 | 3.3 | 90.0 | 160.2 | 1.8 |
|  | 162-17 | M | 6 | 8.4 | 7.6 | 3.4 | 232.3 | 338.0 | 1.5 | 2.8 | 42.3 | 61.0 | 1.4 | 3.4 | 100.8 | 192.1 | 1.9 |

Supplemental Table 1. Summary of monkeys, challenge doses and fever responses

a Virus = isolate used; Age: years; Weight: kg; Dose: log_10_ pfu

b Rmax = maximum residual difference between actual and predicted temperature

c Duration: hours

d Fever-hours: sum of significant temperature elevations, divided by four to convert to hours

e Ave Elev: average elevation, fever-hours / duration

f Overall = fever for the entire post-challenge period

g First = fever during the first fever period, 0.5-2 dpi

h Second = fever during the second fever period, 2.5-8 dpi

* Macaques 5-19 and 6-19 were electively euthanized on day 6 to collect tissue samples for evaluation of viral penetration of the CNS during the second febrile period
