# Supplementary material for: Long-term persistence of viral RNA and inflammation in the CNS of macaques exposed to aerosolized Venezuelan equine encephalitis virus
Source: PLoS Pathog. 2022 Jun 13;18(6):e1009946. doi: 10.1371/journal.ppat.1009946 (PMC9232170; doi:10.1371/journal.ppat.1009946)
Supplement: S2 Table — (DOCX) [file ppat.1009946.s002.docx]

| Table 1. Primer sets used to generate amplicons for Sanger sequencing. | | |
| --- | --- | --- |
| Amplicon | Sense Primer 5’-3’ | Antisense Primer 5’-3’ |
| 1 | nt8-S; GCGCAAGAGAGAAGCCCAAACCAATTACCT | nt2055; TTCTTTCTTGACGCATTGTTTCCTGTCGAT |
| 2 | nt1593; TTTGAGGAGCCCACTCTGGAAGCCGATGTC | nt3882; CTCTTCATGTGAGGATTTCGGTTTGCATAC |
| 3 | nt3717; TATCAGCAGTGTGAAGACCACGCCATTAAG | nt6152; GTGTCTAAGCAACAAGAAGCGCCGTCAACC |
| 4 | nt5680; AATGACGGTTTGACGCGGGTGCATACATCT | nt8034; TCGTCTTAAGTGCGGCCAGAACGTCGTTGT |
| 5 | nt7949; ATTATGCTGGAAGGGAAGATTAACGGCTAC | nt10329; CAGCATGATCCGCAAGGCAGTCGTCAGATT |
| 6 | nt8331;GTGAAGTATACTCCGGAGAACTGCGAGCAAT | nt10863; GTGAACAAGGCGTCGGGAATGT |
| 7 | nt10030; AGCGGGAATCTCGTATAACAC | nt11328; TTGCCAATTGCTGCTATGTTC |
| 8 | nt11119; TGTCACGTGCAAAGGTGATTG | nt11444; TTTTTTTTTTTTTTTTTTTTG |

Sanger sequencing was performed by the Genomics Research Core Facility at the University of Pittsburgh.
