# Supplementary material for: Long-term persistence of viral RNA and inflammation in the CNS of macaques exposed to aerosolized Venezuelan equine encephalitis virus
Source: PLoS Pathog. 2022 Jun 13;18(6):e1009946. doi: 10.1371/journal.ppat.1009946 (PMC9232170; doi:10.1371/journal.ppat.1009946)
Supplement: S1 Data — (PDF) [file ppat.1009946.s003.pdf]

```
Majority            ATGGGCGGCGCAAGAGAGAAGCCCAAACCAATTACCTACCCAAAATGGAGAAAGTTCACGTTGACATCGAGGAAGACAGCCCATTCCTCAGAGCTTTACA
VEEV INH9813-K3E    ....................................................................................................    100
9813-Sequenced      ---------------------...............................................................................     79

Majority            ACGGAGCTTCCCGCAGTTTGAGGTAGAAGCCAAGCAGGTCACTGATAATGACCATGCTAATGCCAGAGCGTTTTCGCATCTGGCTTCAAAACTGATCGAA
VEEV INH9813-K3E    ....................................................................................................    200
9813-Sequenced      ....................................................................................................    179

Majority            ACGGAGGTGGACCCATCCGACACGATCCTTGACATTGGAAGTGCGCCCGCCCGCAGAATGTATTCTAAGCATAAGTATCATTGCATCTGTCCGATGAGAT
VEEV INH9813-K3E    ....................................................................................................    300
9813-Sequenced      ....................................................................................................    279

Majority            GTGCGGAAGATCCGGACAGATTGTACAAGTATGCAACTAAGCTGAAGAAAAATTGCAAGGAAATAACTGACAAGGAATTGGACAAGAAAATGAAGGAGCT
VEEV INH9813-K3E    ....................................................................................................    400
9813-Sequenced      ....................................................................................................    379

Majority            CGCCGCCGTCATGAGCGACCCAGACCTGGAAACTGAGACTATGTGCCTCCACGACGATGAGTCATGTCGCTACGAGGGGCAAGTCGCTGTTTACCAGGAT
VEEV INH9813-K3E    ....................................................................................................    500
9813-Sequenced      ....................................................................................................    479

Majority            GTATACGCAGTTGACGGACCGACAAGTCTCTATCACCAAGCCAACAAGGGAGTTAGAGTCGCCTACTGGATAGGCTTTGACACCACCCCTTTTATGTTTA
VEEV INH9813-K3E    ....................................................................................................    600
9813-Sequenced      ....................................................................................................    579

Majority            AGAACTTGGCTGGAGCATATCCATCATACTCTACCAACTGGGCCGACGAAACCGTGTTAACGGCTCGTAACATAGGCCTATGCAGCTCCGACGTCATGGA
VEEV INH9813-K3E    ....................................................................................................    700
9813-Sequenced      ....................................................................................................    679

Majority            GCGGTCACGTAGAGGGATGTCCATTCTTAGGAAGAAGTATTTGAAACCATCCAATAATGTCCTATTCTCTGTTGGCTCGACCATCTACCACGAGAAGAGG
VEEV INH9813-K3E    ....................................................................................................    800
9813-Sequenced      ....................................................................................................    779

Majority            GACTTACTGAGGAGCTGGCACCTGCCGTCTGTATTTCACTTACGTGGCAAGCAAAATTACACATGTCGGTGTGAGACTATAGTTAGTTGCGACGGGTACG
VEEV INH9813-K3E    ....................................................................................................    900
9813-Sequenced      ....................................................................................................    879

Majority            TCGTTAAAAGAATAGCTATCAGTCCAGGCCTGTATGGGAAGCCTTCAGGCTATGCTGCTACGATGCACCGCGAGGGATTCTTGTGCTGCAAAGTGACAGA
VEEV INH9813-K3E    ....................................................................................................   1000
9813-Sequenced      ....................................................................................................    979

Majority            CACATTGAACGGGGAGAGGGTCTCTTTTCCCGTGTGCACGTATGTGCCAGCTACATTGTGTGACCAAATGACTGGCATACTGGCAACAGATGTCAGTGCG
VEEV INH9813-K3E    ....................................................................................................   1100
9813-Sequenced      ....................................................................................................   1079

Majority            GACGACGCGCAAAAACTGCTGGTTGGGCTCAACCAGCGCATAGTCGTCAACGGTCGCACCCAAAGAAACACCAATACCATGAAGAATTATCTTTTGCCCG
VEEV INH9813-K3E    ....................................................................................................   1200
9813-Sequenced      ....................................................................................................   1179

Majority            TAGTGGCCCAGGCATTTGCTAGGTGGGCAAAGGAATATAAGGAAGATCAAGAAGATGAGAGGCCACTAGGACTACGAGATAGACAGTTAGTCATGGGGTG
VEEV INH9813-K3E    ....................................................................................................   1300
9813-Sequenced      ....................................................................................................   1279

Majority            CTGCTGGGCTTTTAGAAGGCACAAGATAACATCTATTTATAAGCGCCCAGATACCCAAACCATCATCAAAGTGAACAGCGAATTCCACTCATTCGTGCTG
VEEV INH9813-K3E    ....................................................................................................   1400
9813-Sequenced      ....................................................................................................   1379

Majority            CCCAGGATAGGCAGTAACACACTGGAGATCGGGCTGAGAACGAGAATCAGGAAAATGCTAGAAGAGCACAAGGAGCCGTCACCTCTCATTACTGCCGAGG
VEEV INH9813-K3E    ....................................................................................................   1500
9813-Sequenced      ....................................................................................................   1479

Majority            ACATACAAGAGGCTAAGTGCGCAGCCGATGAGGCTAAGGAAGTGCGTGAAGCCGAGGAGCTGCGCGCTGCACTACCACCTTTGGCAGCTGATTTTGAGGA
VEEV INH9813-K3E    ....................................................................................................   1600
9813-Sequenced      ....................................................................................................   1579

Majority            GCCCACTCTGGAAGCCGATGTCGACTTGATGTTACAAGAGGCTGGGGCCGGCTCAGTGGAGACACCTCGTGGCTTGATAAGGGTTACCAGCTATGCCGGC
VEEV INH9813-K3E    ....................................................................................................   1700
9813-Sequenced      ....................................................................................................   1679

Majority            GAGGACAAGATCGGCTCTTACGCAGTGCTTTCTCCACAGGCTGTACTCAAGAGTGAGAAACTATCTTGCATTCACCCTCTCGCTGAACAAGTCATAGTGA
VEEV INH9813-K3E    ....................................................................................................   1800
9813-Sequenced      ....................................................................................................   1779

Majority            TAACACACTCTGGCCGAAAAGGGCGTTATGCCGTGGAACCCTACCATGGAAAAGTAGTGGTGCCAGAGGGACATGCAATACCCGTCCAGGACTTTCAAGC
VEEV INH9813-K3E    ....................................................................................................   1900
9813-Sequenced      ....................................................................................................   1879

Majority            TCTGAGTGAAAGTGCCACCATTGTGTACAACGAACGAGAGTTCGTAAACAGGTACCTGCACCATATTGCCACACATGGAGGAGCGCTGAACACAGATGAA
VEEV INH9813-K3E    ....................................................................................................   2000
```

```
9813-Sequenced     ....................................................................................................     1979

Majority           GAATATTACAAAACTGTCAAGCCCAGCGAGCACGACGGCGAATACCTGTATGACATCGACAGGAAACAATGCGTCAAGAAAGAATTAGTCACTGGGCTAG
VEEV INH9813-K3E   ....................................................................................................     2100
9813-Sequenced     ....................................................................................................     2079

Majority           GGCTTACAGGCGAGCTGGTGGATCCTCCCTTCCATGAATTTGCCTACGAGAGTCTGAGAACACGTCCGGCCGCTCCTTACCAAGTACCAACCATAGGGGT
VEEV INH9813-K3E   ....................................................................................................     2200
9813-Sequenced     ....................................................................................................     2179

Majority           GTATGGCGTGCCGGGGTCAGGCAAGTCTGGCATCATTAAAAGCGCAGTCACCAAAAAAGATCTGGTGGTGAGCGCCAAGAAAGAAAACTGCGCAGAAATA
VEEV INH9813-K3E   ....................................................................................................     2300
9813-Sequenced     ....................................................................................................     2279

Majority           ATAAGGGACGTCAAGAAAATGAAAGGGCTGGACGTCAATGCCAGAACTGTGGACTCAGTGCTCTTGAATGGATGCAAACACCCCGTAGAGACCCTGTATA
VEEV INH9813-K3E   ....................................................................................................     2400
9813-Sequenced     ....................................................................................................     2379

Majority           TTGACGAAGCTTTTGCTTGTCATGCAGGCACTCTCAGAGCGCTCATAGCCATCATAAGACCTAAAAAGGCAGTGCTCTGCGGGGATCCAAAACAGTGTGG
VEEV INH9813-K3E   ....................................................................................................     2500
9813-Sequenced     ....................................................................................................     2479

Majority           CTTTTTCAATATGATGTGCCTGAAAGTGCATTTTAACCACGAGATTTGCACGCAGGTCTTCCACAAAAGCATCTCTCGTCGTTGCACTAAATCCGTGACT
VEEV INH9813-K3E   ....................................................................................................     2600
9813-Sequenced     ....................................................................................................     2579

Majority           TCGGTCGTCTCAACCTTGTTTTATGACAAAAGGATGAGAACGACGAACCCGAAAGAGACTAAGATTGAGATTGACACTACTGGCAGTACCAAACCGAAGC
VEEV INH9813-K3E   ....................................................................................................     2700
9813-Sequenced     ....................................................................................................     2679

Majority           AGGACGATCTCATTCTCACTTGTTTCAGAGGGTGGGTGAAGCAGTTGCAAATAGATTACAAAGGCAACGAAATAATGACGGCAGCTGCCTCTCAAGGGCT
VEEV INH9813-K3E   ....................................................................................................     2800
9813-Sequenced     ....................................................................................................     2779

Majority           GACCCGTAAAGGCGTGTATGCCGTTCGGTACAAGGTGAATGAAAATCCCCTGTACGCACCCACCTCAGAACATGTGAACGTCCTACTGACCCGCACGGAG
VEEV INH9813-K3E   ....................................................................................................     2900
9813-Sequenced     ....................................................................................................     2879

Majority           GACCGTATCGTGTGGAAAACACTAGCCGGTGATCCATGGATAAAAACACTGACGGCCAAGTATCCTGGGAACTTCACTGCCACGATAGAGGAATGGCAAG
VEEV INH9813-K3E   ....................................................................................................     3000
9813-Sequenced     ....................................................................................................     2979

Majority           CAGAGCATGATGCCATCATGAGGCACATCTTGGAGAGACCGGACCCTACCGACGTTTTCCAAAATAAGGCGAACGTGTGTTGGGCCAAGGCTTTGGTGCC
VEEV INH9813-K3E   ....................................................................................................     3100
9813-Sequenced     ....................................................................................................     3079

Majority           GGTACTGAAGACTGCAGGCATAGACATGACCACTGAACAATGGAACACTGTGGATTACTTCGAAACGGACAAAGCTCACTCAGCAGAGATAGTATTGAAC
VEEV INH9813-K3E   ....................................................................................................     3200
9813-Sequenced     ....................................................................................................     3179

Majority           CAACTATGCGTGAGGTTCTTTGGACTCGACCTGGACTCCGGTCTATTTTCTGCACCCACTGTTCCGTTATCCATTAGGAATAATCACTGGGATAATTCCC
VEEV INH9813-K3E   ....................................................................................................     3300
9813-Sequenced     ....................................................................................................     3279

Majority           CGTCGCCTAACATGTACGGGTTGAATAAAGAAGTGGTCCGCCAGCTCTCCCGCAGGTACCCACAACTGCCTCGAGCAGTTGCCACCGGAAGAGTCTATGA
VEEV INH9813-K3E   ....................................................................................................     3400
9813-Sequenced     ....................................................................................................     3379

Majority           CATGAATACTGGCACGCTGCGCAATTATGATCCGCGCATAAATCTAGTACCTGTGAACAGAAGACTGCCTCATGCTTTAGTCCTCCACCATAATGAACAC
VEEV INH9813-K3E   ....................................................................................................     3500
9813-Sequenced     ....................................................................................................     3479

Majority           CCACAGAGTGACTTTTCTTCATTCGTCAGCAAACTGAAGGGCAGAACTGTCTTGGTGGTCGGGGAGAAGTTGTCCGTCCCAGGCAAAAAGGTCGACTGGT
VEEV INH9813-K3E   ....................................................................................................     3600
9813-Sequenced     ....................................................................................................     3579

Majority           TGTCAGACCAGCCTGAGGCTACCTTTAGAGCTCGGCTGGATTTAGGTATCCCAGGTGACGTGCCCAAATACGACATTGTATTTATTAACGTGAGGACTCC
VEEV INH9813-K3E   ....................................................................................................     3700
9813-Sequenced     ....................................................................................................     3679

Majority           ATATAAATACCATCATTATCAGCAGTGTGAAGACCACGCCATTAAGCTTAGTATGTTGACCAAGAAAGCTTGTCTGCATTTGAATCCCGGCGGAACCTGC
VEEV INH9813-K3E   ....................................................................................................     3800
9813-Sequenced     ....................................................................................................     3779

Majority           GTCAGCATAGGTTATGGTTACGCTGACAGGGCCAGCGAGAGCATCATTGGTGCTATAGCGCGGCAGTTCAAGTTCTCCCGGGTATGCAAACCGAAATCCT
VEEV INH9813-K3E   ....................................................................................................     3900
9813-Sequenced     ....................................................................................................     3879

Majority           CACATGAAGAGACAGAAGTACTGTTTGTATTCATTGGGTACGATCGCAAGGCCCGTACGCACAATCCTTACAAGCTTTCATCTACCTTGACCAACATCTA
```

```
VEEV INH9813-K3E ....................................................................................................   4000
9813-Sequenced   ....................................................................................................   3979

Majority         TACAGGTTCCAGACTCCACGAAGCCGGATGCGCACCCTCATATCATGTGGTGCGAGGGGATATTGCCACGGCCACCGAAGGAGTGATCATAAATGCTGCC
VEEV INH9813-K3E ....................................................................................................   4100
9813-Sequenced   ....................................................................................................   4079

Majority         AACAGCAAAGGACAACCTGGCGGAGGGGTGTGCGGAGCGCTGTATAAGAAATTCCCGGAAAGCTTCGATTTACAGCCGATCGAAGTGGGAAAAGCGCGAC
VEEV INH9813-K3E ....................................................................................................   4200
9813-Sequenced   ....................................................................................................   4179

Majority         TGGTCAAAGGTGTAACTAAACATATTATTCATGCCGTAGGGCCCAACTTCAACAAAGTTTCGGAAGTTGAAGGGGACAAACAGTTGGCAGAGGCTTATGA
VEEV INH9813-K3E ....................................................................................................   4300
9813-Sequenced   ....................................................................................................   4279

Majority         GTCCATCGCTAAAATTGTCAACGATAACAATTACAAGTCAGTAGCGATTCCACTGTTGTCCACCGGCATCTTTTCCGGGAACAAAGATCGACTAACCCAA
VEEV INH9813-K3E ....................................................................................................   4400
9813-Sequenced   ....................................................................................................   4379

Majority         TCATTGAACCATTTGCTGACAGCTTTAGACACCACTGATGCAGATGTAGCCATATACTGCAGGGACAAGAAATGGGAAATGACTCTCAAGGAAGCAGTGG
VEEV INH9813-K3E ....................................................................................................   4500
9813-Sequenced   ....................................................................................................   4479

Majority         CTAGGAGAGAAGCAGTGGAGGAGATATGCATATCAGACGACTCTTCGGTGACAGAACCGGATGCAGAGCTGGTGAGGGTACATCCGAAGAGTTCTTTGGC
VEEV INH9813-K3E ....................................................................................................   4600
9813-Sequenced   ....................................................................................................   4579

Majority         TGGAAGGAAGGGCTACAGCACAAGTGATGGCAAGACTTTCTCATATTTGGAAGGGACCAAATTTCACCAGGCGGCCAAGGATATAGCAGAAATTAATGCC
VEEV INH9813-K3E ....................................................................................................   4700
9813-Sequenced   ....................................................................................................   4679

Majority         ATGTGGCCAGTTGCAACGGAGGCCAATGAGCAAGTATGCATGTATATCCTCGGTGAAAGCATGAGCAGCATTAGGTCGAAATGCCCCGTCGAGGAGTCGG
VEEV INH9813-K3E ....................................................................................................   4800
9813-Sequenced   ....................................................................................................   4779

Majority         AAGCCTCCACACCACCTAGCACGCTGCCTTGCTTGTGCATCCATGCTATGACTCCAGAAAGAGTACAACGCCTAAAAGCCTCACGTCCAGAACAAATTAC
VEEV INH9813-K3E ....................................................................................................   4900
9813-Sequenced   ....................................................................................................   4879

Majority         TGTGTGCTCATCCTTTCCATTGCCGAAGTATAGAATCACTGGTGTGCAGAAGATCCAGTGCTCCCAGCCTATACTGTTCTCACCGAAGGTGCCTGCGTAC
VEEV INH9813-K3E ....................................................................................................   5000
9813-Sequenced   ....................................................................................................   4979

Majority         ATTCATCCACGGAAGTACCTCGTGGAAACACCACCGGTAGAAGAGACTCCGGAGTCGCCGGCAGAGAACCAATCCACAGAGGGGACACCTGAACAACCAG
VEEV INH9813-K3E ....................................................................................................   5100
9813-Sequenced   ....................................................................................................   5079

Majority         CACTTGTAAACGTGGATGCAACCAGGACTAGAATGCCTGAACCGATCATCATTGAAGAGGAAGAAGAGGATAGTATAAGTTTGCTGTCAGACGGCCCGAC
VEEV INH9813-K3E ....................................................................................................   5200
9813-Sequenced   ....................................................................................................   5179

Majority         CCACCAGGTGCTGCAAGTCGAGGCAGACATTCACGGGTCGCCTTCTGTATCCAGCTCATCCTGGTCCATTCCTCATGCATCCGACTTTGATGTGGACAGC
VEEV INH9813-K3E ....................................................................................................   5300
9813-Sequenced   ....................................................................................................   5279

Majority         TTATCCATCCTTGACACCCTGGATGGAGCTAGCGTGACCAGCGAGGCAGTGTCAGCCGAGACTAACTCCTACTTCGCAAGGAGCATGGAGTTTCGGGCGC
VEEV INH9813-K3E ....................................................................................................   5400
9813-Sequenced   ....................................................................................................   5379

Majority         GACCGGTGCCTGCGCCTCGAACCGTATTCAGGAACCCTCCACATCCCGCACCGCGCACAAGAACACCGCCACTCGCACACAGCAGGGCCAGCTCGAGAAC
VEEV INH9813-K3E ....................................................................................................   5500
9813-Sequenced   ....................................................................................................   5479

Majority         TAGCCTAGTTTCCACCCCGCCAGGCGTGAATAGGGTGATTACTAGAGAGGAGCTCGAGGCGCTTACCCCGTCCCGCGCTCCTAGCAGGTCGGCCTCAAGA
VEEV INH9813-K3E ....................................................................................................   5600
9813-Sequenced   ....................................................................................................   5579

Majority         ACTAGCCTGGTCTCTAACCCGCCAGGCGTAAATAGGGTGATTACAAGAGAGGAGTTTGAGGCGTTCGTAGCACAACAACAATGACGGTTTGACGCGGGTG
VEEV INH9813-K3E ....................................................................................................   5700
9813-Sequenced   ....................................................................................................   5679

Majority         CATACATCTTTTCCTCCGATACCGGTCAAGGGCATTTACAACAAAAATCAGTAAGGCAAACGGTGTTATCCGAAGTGGTGTTGGAGAGGACCGAATTGGA
VEEV INH9813-K3E ....................................................................................................   5800
9813-Sequenced   ....................................................................................................   5779

Majority         GATTTCGTATGCCCCGCGCCTCGACCAGGAAAAAGAAGAACTACTACGCAAGAAATTACAGCTGAATCCCACACCTGCTAACAGAAGCAGATACCAGTCC
VEEV INH9813-K3E ....................................................................................................   5900
9813-Sequenced   ....................................................................................................   5879
```

```
Majority          AGGAGGGTGGAGAATATGAAAGCCATAACAGCTAGACGTATTCTGCAAGGCCTAGGGCATTATTTGAAGGCAGAAGGAAAAGTGGAGTGCTATCGAACCC
VEEV INH9813-K3E  ....................................................................................................   6000
9813-Sequenced    ....................................................................................................   5979

Majority          TGCATCCTGTTCCTTTGTATTCATCTAGTGTGAATCGTGCTTTTTCAAGCCCCAAGGTCGCAGTGGAAGCCTGCAATGCCATGCTGAAAGAAAATTTTCC
VEEV INH9813-K3E  ....................................................................................................   6100
9813-Sequenced    ....................................................................................................   6079

Majority          GACTGTAGCTTCCTACTGTATTATTCCAGAGTACGATGCCTATCTGGACATGGTTGACGGCGCTTCTTGTTGCTTAGACACTGCCAGTTTTTGCCCTGCG
VEEV INH9813-K3E  ....................................................................................................   6200
9813-Sequenced    ....................................................................................................   6179

Majority          AAGCTGCGCAGCTTTCCAAAGAAACACTCCTATTTGGAACCCACAATACGGTCGGCAGTGCCATCAGCGATTCAGAACACGCTCCAGAACGTCCTGGCAG
VEEV INH9813-K3E  ....................................................................................................   6300
9813-Sequenced    ....................................................................................................   6279

Majority          CTGCCACAAAAAGAAATTGCAACGTCACGCAAATGAGAGAATTGCCCGTATTGGATTCGGCTGCCTTTAATGTGGAATGCTTCAAGAAATATGCGTGCAA
VEEV INH9813-K3E  ....................................................................................................   6400
9813-Sequenced    ....................................................................................................   6379

Majority          TAATGAATATTGGGAAACGTTTAAAGAAAACCCCATCAGGCTTACTGAAGAAAATGTGGTAAATTACATTACTAAATTAAAAGGACCAAAAGCTGCTGCT
VEEV INH9813-K3E  ....................................................................................................   6500
9813-Sequenced    ....................................................................................................   6479

Majority          CTTTTTGCGAAGACACATAATTTGAATATGTTACAGGACATACCAATGGACAGGTTTGTAATGGACTTAAAGAGGGACGTGAAAGTGACTCCAGGAACAA
VEEV INH9813-K3E  ....................................................................................................   6600
9813-Sequenced    ....................................................................................................   6579

Majority          AACATACTGAAGAACGGCCCAAGGTACAGGTGATTCAGGCTGCCGATCCACTAGCGACAGCGTATCTGTGCGGAATCCACCGGGAGTTGGTTAGGAGATT
VEEV INH9813-K3E  ....................................................................................................   6700
9813-Sequenced    ....................................................................................................   6679

Majority          AAATGCTGTCCTGCTTCCGAACATCCATACACTGTTTGACATGTCGGCTGAAGACTTTGACGCTATTATTGCCGAGCATTTCCAGCCTGGGGACTGTGTA
VEEV INH9813-K3E  ....................................................................................................   6800
9813-Sequenced    ....................................................................................................   6779

Majority          CTGGAAACTGACATTGCGTCGTTTGATAAAAGTGAGGACGACGCCATGGCTCTGACCGCGTTAATGATTCTGGAAGACCTAGGAGTGGACGCAGAGCTGT
VEEV INH9813-K3E  ....................................................................................................   6900
9813-Sequenced    ....................................................................................................   6879

Majority          TGACGCTGATTGAGGCGGCTTTCGGCGAAATATCATCAATACATTTGCCCACCAAAACTAAATTTAAATTCGGAGCCATGATGAAATCCGGAATGTTCCT
VEEV INH9813-K3E  ....................................................................................................   7000
9813-Sequenced    ....................................................................................................   6979

Majority          CACACTGTTTGTGAACACAGTCATCAACATCGTAATCGCAAGCAGAGTGTTAAGAGAGCGGCTAACCGGATCACCATGTGCAGCATTCATTGGAGATGAC
VEEV INH9813-K3E  ....................................................................................................   7100
9813-Sequenced    ....................................................................................................   7079

Majority          AATATCGTGAAAGGAGTCAAATCTGACAAATTAATGGCAGACAGGTGCGCCACTTGGTTGAACATGGAAGTCAAGATCATAGACGCCGTGGTGGGCGAGA
VEEV INH9813-K3E  ....................................................................................................   7200
9813-Sequenced    ....................................................................................................   7179

Majority          AAGCGCCCTATTTTTGTGGAGGGTTTATCTTGTGTGACTCCGTGACCGGCACAGCGTGCCGTGTGGCAGACCCCCTAAAAAGGCTGTTTAAGCTTGGCAA
VEEV INH9813-K3E  ....................................................................................................   7300
9813-Sequenced    ....................................................................................................   7279

Majority          ACCCCTGGCAGTAGACGATGAACATGACGATGACAGGAGAAGGGCATTACACGAAGAGTCAACACGCTGGAATCGAGTGGGAATTCTTCCAGAGCTGTGT
VEEV INH9813-K3E  ....................................................................................................   7400
9813-Sequenced    ....................................................................................................   7379

Majority          AAGGCAGTAGAATCAAGGTATGAAACCGTAGGAACTTCCATCATAGTTATGGCCATGACTACTCTAGCTAGCAGTGTTAAATCATTCAGCTACCTGAGAG
VEEV INH9813-K3E  ....................................................................................................   7500
9813-Sequenced    ....................................................................................................   7479

Majority          GGGCCCCTATAACTCTCTACGGCTAACCTGAATGGACTACGACATAGTCTAGTCCGCCAAGATGTTCCCGTTCCAACCAATGTATCCGATGCAGCCAATG
VEEV INH9813-K3E  ....................................................................................................   7600
9813-Sequenced    ....................................................................................................   7579

Majority          CCCTATCGTAACCCGTTCGCGGCCCCGCGCAGGCCCTGGTTCCCCAGAACCGACCCTTTTCTGGCGATGCAGGTGCAGGAATTAACCCGCTCGATGGCTA
VEEV INH9813-K3E  ....................................................................................................   7700
9813-Sequenced    ....................................................................................................   7679

Majority          ACCTGACGTTCAAGCAACGCCGGGACGCGCCACCTGAGGGGCCACCTGCTAAGAAACCTAAGAGGGAGGCCCCGCAAAAGCAAAAAGGGGGAGGCCAAGG
VEEV INH9813-K3E  ....................................................................................................   7800
9813-Sequenced    ....................................................................................................   7779

Majority          GAAGAAGAAGAAGAACCAGGGGAAGAAGAAGGCCAAGACGGGGCCGCCTAATCCGAAGGCACAGAGTGGAAACAAGAAGAAGCCCAACAAGAAACCAGGC
VEEV INH9813-K3E  ....................................................................................................   7900
9813-Sequenced    ....................................................................................................   7879
```

```
Majority        AAGAGACAGCGCATGGTCATGAAATTGGAATCTGACAAGACATTCCCAATTATGCTGGAAGGGAAGATTAACGGCTACGCTTGCGTGGTCGGCGGGAAGT
VEEV INH9813-K3E ....................................................................................................  8000
9813-Sequenced  ....................................................................................................  7979

Majority        TATTCAGGCCGATGCACGTGGAAGGCAAGATCGACAACGACGTTCTGGCCGCACTTAAGACGAAGAAAGCATCCAAATATGATCTTGAGTATGCAGATGT
VEEV INH9813-K3E ....................................................................................................  8100
9813-Sequenced  ....................................................................................................  8079

Majority        GCCACAGAACATGCGGGCCGATACATTCAAGTACACCCATGAGAAGCCCCAAGGCTATTACAGCTGGCATCATGGAGCAGTCCAATATGAAAATGGGCGT
VEEV INH9813-K3E ....................................................................................................  8200
9813-Sequenced  ....................................................................................................  8179

Majority        TTCACGGTGCCAAAAGGAGTTGGGGCCAAGGGAGACAGCGGAAGACCCATTCTGGATAATCAGGGACGGGTGGTCGCTATTGTGCTGGGAGGTGTGAATG
VEEV INH9813-K3E ....................................................................................................  8300
9813-Sequenced  ....................................................................................................  8279

Majority        AAGGATCTAGGACAGCCCTTTCAGTCGTCATGTGGAACGAGAAGGGAGTAACTGTGAAGTATACTCCGGAGAACTGCGAGCAATGGTCACTAGTGACCAC
VEEV INH9813-K3E ....................................................................................................  8400
9813-Sequenced  ....................................................................................................  8379

Majority        TATGTGCCTGCTCGCCAATGTGACGTTCCCATGTGCCGAACCACCAATTTGCTACGACAGAAAACCAGCAGAGACTTTGGCCATGCTCAGCGTTAACGTT
VEEV INH9813-K3E ....................................................................................................  8500
9813-Sequenced  ....................................................................................................  8479

Majority        GACAACCCGGGCTACGATGAGCTACTGGAAGCAGCTGTTAAGTGCCCCGGAAGAAAAAGGAGATCTACCGAGGAGCTGTTTAAGGAGTATAAGCTAACGC
VEEV INH9813-K3E ....................................................................................................  8600
9813-Sequenced  ....................................................................................................  8579

Majority        GCCCTTACATGGCCAGATGCATCAGATGTGCCGTTGGGAGCTGCCATAGTCCAATAGCAATTGAGGCAGTGAAGAGCGACGGGCACGACGGCTATGTTAG
VEEV INH9813-K3E ....................................................................................................  8700
9813-Sequenced  ....................................................................................................  8679

Majority        ACTTCAGACTTCCTCGCAGTATGGCCTGGATTCCTCTGGCAACTTAAAGGGAAGGACTATGCGGTATGATATGCACGGGACCATTGAAGAGATACCACTA
VEEV INH9813-K3E ....................................................................................................  8800
9813-Sequenced  ....................................................................................................  8779

Majority        CATCAAGTGTCACTCCACACATCTCGCCCGTGTCACATTGTGGATGGGCATGGTTATTTTCTGCTTGCTAGGTGCCCGGCAGGGGACTCCATCACCATGG
VEEV INH9813-K3E ....................................................................................................  8900
9813-Sequenced  ....................................................................................................  8879

Majority        AATTTAAGAAAGGTTCAGTCACACACTCCTGCTCAGTGCCGTATGAAGTGAAATTTAATCCTGTAGGCAGAGAACTCTACACTCATCCACCAGAACACGG
VEEV INH9813-K3E ....................................................................................................  9000
9813-Sequenced  ....................................................................................................  8979

Majority        AGCAGAGCAAGCGTGCCAAGTCTACGCGCACGATGCACAGAACAGAGGAGCTTATGTCGAGATGCACCTCCCGGGCTCAGAAGTGGACAGCAGTTTGATT
VEEV INH9813-K3E ....................................................................................................  9100
9813-Sequenced  ....................................................................................................  9079

Majority        TCCTTGAGCGGCAGTTCAGTCACCGTGACACCTCCTGTCGGGACTAGCGCCTTGGTGGAATGCAAGTGCGGCGGCACAAAGATCTCCGAAACCATCAACA
VEEV INH9813-K3E ....................................................................................................  9200
9813-Sequenced  ....................................................................................................  9179

Majority        AGGCAAAACAGTTCAGCCAGTGCACAAAGAAGGAGCAGTGCAGAGCATATCGACTGCAGAATGACAAGTGGGTGTATAATTCTGACAAACTGCCCAAAGC
VEEV INH9813-K3E ....................................................................................................  9300
9813-Sequenced  ....................................................................................................  9279

Majority        AGCGGGAGCCACCCTAAAAGGAAAACTACACGTCCCGTTCTTGCTGGCAGACGGCAAATGCACCGTGCCTCTAGCACCGGAACCTATGATAACCTTCGGT
VEEV INH9813-K3E ....................................................................................................  9400
9813-Sequenced  ....................................................................................................  9379

Majority        TTCCGATCAGTGTCACTGAAACTGCACCCTAAGAATCCCACATATCTGACCACTCGCCAACTTGCTGATGAGCCTCATTACACGCACGAGCTCATATCTG
VEEV INH9813-K3E ....................................................................................................  9500
9813-Sequenced  ....................................................................................................  9479

Majority        AACCAGCTGTTAGGAATTTTACCGTCACTGAAAAGGGGTGGGAGTTTGTATGGGGAAACCATCCGCCGAAAAGGTTTTGGGCACAGGAAACAGCACCCGG
VEEV INH9813-K3E ....................................................................................................  9600
9813-Sequenced  ....................................................................................................  9579

Majority        AAATCCACATGGGCTGCCACATGAGGTGATAACTCATTATTACCACAGATACCCTATGTCCACCATCCTGGGTTTGTCAATTTGCGCCGCCATTGTAACC
VEEV INH9813-K3E ....................................................................................................  9700
9813-Sequenced  ....................................................................................................  9679

Majority        GTTTCCGTTGCAGCGTCCACCTGGCTGTTTTGCAAATCCAGAGTTTCGTGCCTAACTCCTTACCGGCTAACACCTAACGCCAGGATGCCGCTTTGCCTGG
VEEV INH9813-K3E ....................................................................................................  9800
9813-Sequenced  ....................................................................................................  9779

Majority        CCGTGCTTTGCTGCGCCCGCACTGCCCGGGCCGAGACCACCTGGGAGTCCTTGGATCACCTATGGAACAATAACCAACAGATGTTCTGGATTCAATTGCT
VEEV INH9813-K3E ....................................................................................................  9900
```

```
9813-Sequenced     ....................................................................................................   9879

Majority           GATCCCTCTGGCCGCCTTGATTGTAGTGACTCGCCTGCTCAAGTGCGTGTGCTGTGTAGTGCCTTTTTAGTCGTGGCCGGCGCCGCAGGCGCCGGCGCC
VEEV INH9813-K3E   ....................................................................................................   10000
9813-Sequenced     ....................................................................................................   9979

Majority           TACGAGCACGCGACCACGATGCCGAGCCAAGCGGGAATCTCGTATAACACCATAGTCAACAGAGCAGGCTACGCGCCACTCCCTATCAGCATAACACCAA
VEEV INH9813-K3E   ....................................................................................................   10100
9813-Sequenced     ....................................................................................................   10079

Majority           CAAAGATCAAGCTGATACCCACAGTGAACTTGGAGTACGTCACCTGCCACTACAAAACAGGAATGGATTCACCAGCCATCAAATGCTGCGGATCTCAGGA
VEEV INH9813-K3E   ....................................................................................................   10200
9813-Sequenced     ....................................................................................................   10179

Majority           ATGTACTCCAACTAACAGGCCTGATGAACAGTGCAAAGTCTTCACAGGGGTTTACCCGTTCATGTGGGGAGGTGCATATTGCTTTTGCGACACTGAGAAT
VEEV INH9813-K3E   ....................................................................................................   10300
9813-Sequenced     ....................................................................................................   10279

Majority           ACTCAGGTCAGCAAGGCCTACGTAATGAAATCTGACGACTGCCTTGCGGATCATGCTGAAGCATACAAAGCGCACACAGCCTCAGTGCAGGCGTTCCTCA
VEEV INH9813-K3E   ....................................................................................................   10400
9813-Sequenced     ....................................................................................................   10379

Majority           ACATCACAGTGGGGGAACACTCTATTGTGACCACCGTGTATGTGAATGGAGAAACTCCTGTGAACTTCAATGGGGTCAAACTAACTGCAGGTCCACTTTC
VEEV INH9813-K3E   ....................................................................................................   10500
9813-Sequenced     ....................................................................................................   10479

Majority           CACAGCTTGGACACCCTTTGACAGAAAAATCGTGCAGTATGCCGGGGAGATCTATAATTACGATTTTCCTGAGTATGGGGCAGGACAACCAGGAGCATTT
VEEV INH9813-K3E   ....................................................................................................   10600
9813-Sequenced     ....................................................................................................   10579

Majority           GGAGACATACAATCCAGAACAGTCTCAAGCTCAGATCTGTATGCCAATACCAACCTAGTGCTGCAGAGACCCAAAGCAGGAGCGATCCATGTGCCATACA
VEEV INH9813-K3E   ....................................................................................................   10700
9813-Sequenced     ....................................................................................................   10679

Majority           CTCAGGCACCATCGGGTTTTGAGCAATGGAAGAAAGATAAAGCTCCGTCATTGAAATTCACCGCCCCTTTCGGATGCGAAATATATACAAACCCCATTCG
VEEV INH9813-K3E   ....................................................................................................   10800
9813-Sequenced     ....................................................................................................   10779

Majority           CGCCGAAAATTGTGCTGTAGGGTCAATTCCATTAGCCTTTGACATTCCCGACGCCTTGTTCACCAGGGTGTCAGAAACACCGACACTTTCAGCGGCCGAA
VEEV INH9813-K3E   ....................................................................................................   10900
9813-Sequenced     ....................................................................................................   10879

Majority           TGCACTCTTAACGAGTGCGTGTATTCATCCGACTTTGGCGGGATCGCCACGGTCAAGTATTCGGCCAGCAAGTCAGGCAAGTGCGCAGTCCATGTGCCAT
VEEV INH9813-K3E   ....................................................................................................   11000
9813-Sequenced     ....................................................................................................   10979

Majority           CAGGGACTGCTACCCTAAAAGAAGCAGCAGTCGAGCTAACCGAGCAAGGGTCGGCGACCATTCATTTCTCGACCGCAAATATCCACCCGGAGTTCAGGCT
VEEV INH9813-K3E   ....................................................................................................   11100
9813-Sequenced     ....................................................................................................   11079

Majority           CCAAATATGCACATCATATGTCACGTGCAAAGGTGATTGTCACCCCCCGAAAGACCACATTGTGACACACCCCCAGTATCACGCCCAAACATTTACAGCC
VEEV INH9813-K3E   ....................................................................................................   11200
9813-Sequenced     ....................................................................................................   11179

Majority           GCGGTGTCAAAAACCGCGTGGACGTGGTTAACATCCCTGCTGGGAGGATCGGCCGTAATTATTATAATTGGCTTAGTGCTGGCTACTATTGTGGCCATGT
VEEV INH9813-K3E   ....................................................................................................   11300
9813-Sequenced     ....................................................................................................   11279

Majority           ACGTGCTGACCAACCAGAAACATAATTGAACATAGCAGCAATTGGCAAGCTGCTTATATAGAACTTGCGGCGATTGGCATGCCGCTTTAAAATTTTATTT
VEEV INH9813-K3E   ....................................................................................................   11400
9813-Sequenced     ....................................................................................................   11379

Majority           TATTTTCTTTTCTTTTCCGAATCGGATTTGTTTTTAATATTTCAAAAAAAAAAAAAAAAAAAAAGGGTACXXXXXXXXXXXXXXXXXXXXXXXXXXXXXX
VEEV INH9813-K3E   .......................................................................GCGGCCGCAAGTACCGAGCTCGAATTCGT   11500
9813-Sequenced     .................................................................AAAA.A                                 11450

Majority           XXXXXXXXXXXXXXXXXXXXXXXXXXXXXXXXXXXXXXXXXXXXXXXXXXXXXXXXXXXXXXXXXXXXXXXXXXXXXXXXXXXXXXXXXXXXXXXXXXXX
VEEV INH9813-K3E   AATCATGGTCATAGCTGTTTCCTGTGTGAAATTGTTATCCGCTCACAATTCCACACAACATACGAGCCGGAAGCATAAAGTGTAAAGCCTGGGGTGCCTA   11600
9813-Sequenced                                                                                                            11450

Majority           XXXXXXXXXXXXXXXXXXXXXXXXXXXXXXXXXXXXXXXXXXXXXXXXXXXXXXXXXXXXXXXXXXXXXXXXXXXXXXXXXXXXXXXXXXXXXXXXXXXX
VEEV INH9813-K3E   ATGAGTGAGCTAACTCACATTAATTGCGTTGCGCTCACTGCCCGCTTTCCAGTCGGGAAACCTGTCGTGCCAGTCGAGGGGAATTAATTCTTGAAGACGA   11700
9813-Sequenced                                                                                                            11450

Majority           XXXXXXXXXXXXXXXXXXXXXXXXXXXXXXXXXXXXXXXXXXXXXXXXXXXXXXXXXXXXXXXXXXXXXXXXXXXXXXXXXXXXXXXXXXXXXXXXXXXX
VEEV INH9813-K3E   AAGGGCCAGGTGGCACTTTTCGGGGAAATGTGCGCGGAACCCCTATTTGTTTATTTTTCTAAATACATTCAAATATGTATCCGCTCATGAGACAATAACC   11800
9813-Sequenced                                                                                                            11450

Majority           XXXXXXXXXXXXXXXXXXXXXXXXXXXXXXXXXXXXXXXXXXXXXXXXXXXXXXXXXXXXXXXXXXXXXXXXXXXXXXXXXXXXXXXXXXXXXXXXXXXX
```

```
VEEV INH9813-K3E CTGATAAATGCTTCAATAATATTGAAAAAGGAAGAGTATGAGTATTCAACATTTCCGTGTCGCCCTTATTCCCTTTTTTGCGGCATTTTGCCTTCCTGTT     11900
9813-Sequenced                                                                                                         11450

Majority         XXXXXXXXXXXXXXXXXXXXXXXXXXXXXXXXXXXXXXXXXXXXXXXXXXXXXXXXXXXXXXXXXXXXXXXXXXXXXXXXXXXXXXXXXXXXXXXXXXXX
VEEV INH9813-K3E TTTGCTCACCCAGAAACGCTGGTGAAAGTAAAAGATGCTGAAGATCAGTTGGGTGCACGCGTGGGTTACATCGAACTGGATCTCAACAGCGGTAAGATCC     12000
9813-Sequenced                                                                                                         11450

Majority         XXXXXXXXXXXXXXXXXXXXXXXXXXXXXXXXXXXXXXXXXXXXXXXXXXXXXXXXXXXXXXXXXXXXXXXXXXXXXXXXXXXXXXXXXXXXXXXXXXXX
VEEV INH9813-K3E TTGAGAGTTTTCGCCCCGAAGAACGTTTTCCAATGATGAGCACTTTTAAAGTTCTGCTATGTGGCGCGGTATTATCCCGTGTTGACGCCGGGCAAGAGCA     12100
9813-Sequenced                                                                                                         11450

Majority         XXXXXXXXXXXXXXXXXXXXXXXXXXXXXXXXXXXXXXXXXXXXXXXXXXXXXXXXXXXXXXXXXXXXXXXXXXXXXXXXXXXXXXXXXXXXXXXXXXXX
VEEV INH9813-K3E ACTCGGTCGCCGCATACACTATTCTCAGAATGACTTGGTTGAGTACTCACCAGTCACAGAAAAGCATCTTACGGATGGCATGACAGTAAGAGAATTATGC     12200
9813-Sequenced                                                                                                         11450

Majority         XXXXXXXXXXXXXXXXXXXXXXXXXXXXXXXXXXXXXXXXXXXXXXXXXXXXXXXXXXXXXXXXXXXXXXXXXXXXXXXXXXXXXXXXXXXXXXXXXXXX
VEEV INH9813-K3E AGTGCTGCCATAACCATGAGTGATAACACTGCGGCCAACTTACTTCTGACAACGATCGGAGGACCGAAGGAGCTAACCGCTTTTTTGCACAACATGGGGG     12300
9813-Sequenced                                                                                                         11450

Majority         XXXXXXXXXXXXXXXXXXXXXXXXXXXXXXXXXXXXXXXXXXXXXXXXXXXXXXXXXXXXXXXXXXXXXXXXXXXXXXXXXXXXXXXXXXXXXXXXXXXX
VEEV INH9813-K3E ATCATGTAACTCGCCTTGATCGTTGGGAACCGGAGCTGAATGAAGCCATACCAAACGACGAGCGTAACACCACGATGCCTGTAGCAATGGCAACAACGTT     12400
9813-Sequenced                                                                                                         11450

Majority         XXXXXXXXXXXXXXXXXXXXXXXXXXXXXXXXXXXXXXXXXXXXXXXXXXXXXXXXXXXXXXXXXXXXXXXXXXXXXXXXXXXXXXXXXXXXXXXXXXXX
VEEV INH9813-K3E GCGCAAACTATTAACTGGCGAACTACTTACTCTAGCTTCCCGGCAACAATTAATAGACTGGATGGAGGCGGATAAAGTTGCAGGACCACTTCTGCGCTCG     12500
9813-Sequenced                                                                                                         11450

Majority         XXXXXXXXXXXXXXXXXXXXXXXXXXXXXXXXXXXXXXXXXXXXXXXXXXXXXXXXXXXXXXXXXXXXXXXXXXXXXXXXXXXXXXXXXXXXXXXXXXXX
VEEV INH9813-K3E GCCCTTCCGGCTGGCTGGTTTATTGCTGATAAATCTGGAGCCGGTGAGCGTGGGTCTCGCGGTATCATTGCAGCACTGGGGCCAGATGGTAAGCCCTCCC     12600
9813-Sequenced                                                                                                         11450

Majority         XXXXXXXXXXXXXXXXXXXXXXXXXXXXXXXXXXXXXXXXXXXXXXXXXXXXXXXXXXXXXXXXXXXXXXXXXXXXXXXXXXXXXXXXXXXXXXXXXXXX
VEEV INH9813-K3E GTATCGTAGTTATCTACACGACGGGGAGTCAGGCAACTATGGATGAACGAAATAGACAGATCGCTGAGATAGGTGCCTCACTGATTAAGCATTGGTAACT     12700
9813-Sequenced                                                                                                         11450

Majority         XXXXXXXXXXXXXXXXXXXXXXXXXXXXXXXXXXXXXXXXXXXXXXXXXXXXXXXXXXXXXXXXXXXXXXXXXXXXXXXXXXXXXXXXXXXXXXXXXXXX
VEEV INH9813-K3E GTCAGACCAAGTTTACTCATATATACTTTAGATTGATTTAAAACTTCATTTTTAATTTAAAAGGATCTAGGTGAAGATCCTTTTTGATAATCTCATGACC     12800
9813-Sequenced                                                                                                         11450

Majority         XXXXXXXXXXXXXXXXXXXXXXXXXXXXXXXXXXXXXXXXXXXXXXXXXXXXXXXXXXXXXXXXXXXXXXXXXXXXXXXXXXXXXXXXXXXXXXXXXXXX
VEEV INH9813-K3E AAAATCCCTTAACGTGAGTTTTCGTTCCACTGAGCGTCAGACCCCGTAGAAAAGATCAAAGGATCTTCTTGAGATCCTTTTTTTCTGCGCGTAATCTGCT     12900
9813-Sequenced                                                                                                         11450

Majority         XXXXXXXXXXXXXXXXXXXXXXXXXXXXXXXXXXXXXXXXXXXXXXXXXXXXXXXXXXXXXXXXXXXXXXXXXXXXXXXXXXXXXXXXXXXXXXXXXXXX
VEEV INH9813-K3E GCTTGCAAACAAAAAAACCACCGCTACCAGCGGTGGTTTGTTTGCCGGATCAAGAGCTACCAACTCTTTTTCCGAAGGTAACTGGCTTCAGCAGAGCGCA     13000
9813-Sequenced                                                                                                         11450

Majority         XXXXXXXXXXXXXXXXXXXXXXXXXXXXXXXXXXXXXXXXXXXXXXXXXXXXXXXXXXXXXXXXXXXXXXXXXXXXXXXXXXXXXXXXXXXXXXXXXXXX
VEEV INH9813-K3E GATACCAAATACTGTCCTTCTAGTGTAGCCGTAGTTAGGCCACCACTTCAAGAACTCTGTAGCACCGCCTACATACCTCGCTCTGCTAATCCTGTTACCA     13100
9813-Sequenced                                                                                                         11450

Majority         XXXXXXXXXXXXXXXXXXXXXXXXXXXXXXXXXXXXXXXXXXXXXXXXXXXXXXXXXXXXXXXXXXXXXXXXXXXXXXXXXXXXXXXXXXXXXXXXXXXX
VEEV INH9813-K3E GTGGCTGCTGCCAGTGGCGATAAGTCGTGTCTTACCGGGTTGGACTCAAGACGATAGTTACCGGATAAGGCGCAGCGGTCGGGCTGAACGGGGGGTTCGT     13200
9813-Sequenced                                                                                                         11450

Majority         XXXXXXXXXXXXXXXXXXXXXXXXXXXXXXXXXXXXXXXXXXXXXXXXXXXXXXXXXXXXXXXXXXXXXXXXXXXXXXXXXXXXXXXXXXXXXXXXXXXX
VEEV INH9813-K3E GCACACAGCCCAGCTTGGAGCGAACGACCTACACCGAACTGAGATACCTACAGCGTGAGCATTGAGAAAGCGCCACGCTTCCCGAAGGGAGAAAGGCGGA     13300
9813-Sequenced                                                                                                         11450

Majority         XXXXXXXXXXXXXXXXXXXXXXXXXXXXXXXXXXXXXXXXXXXXXXXXXXXXXXXXXXXXXXXXXXXXXXXXXXXXXXXXXXXXXXXXXXXXXXXXXXXX
VEEV INH9813-K3E CAGGTATCCGGTAAGCGGCAGGGTCGGAACAGGAGAGCGCACGAGGGAGCTTCCAGGGGGAAACGCCTGGTATCTTTATAGTCCTGTCGGGTTTCGCCAC     13400
9813-Sequenced                                                                                                         11450

Majority         XXXXXXXXXXXXXXXXXXXXXXXXXXXXXXXXXXXXXXXXXXXXXXXXXXXXXXXXXXXXXXXXXXXXXXXXXXXXXXXXXXXXXXXXXXXXXXXXXXXX
VEEV INH9813-K3E CTCTGACTTGAGCGTCGATTTTTGTGATGCTCGTCAGGGGGGCGGAGCCTATGGAAAAACGCCAGCAACGCGAGCTCTGGCGAAAGGGGGATGTGCTGCA     13500
9813-Sequenced                                                                                                         11450

Majority         XXXXXXXXXXXXXXXXXXXXXXXXXXXXXXXXXXXXXXXXXXXXXXXXXXXXXXXXXXXXXXXXXXXXXXXXXXXXXXXXXXXXXXXXXXXXXXXXXXXX
VEEV INH9813-K3E AGGCGATTAAGTTGGGTAACGCCAGGGTTTTCCCAGTCACGACGTTGTAAAACGACGGCCAGTGCCAAGCTTGCATGCCTGCAGGTCGACTCTAGAGGAT     13600
9813-Sequenced                                                                                                         11450

Majority         XXXXXXXXXXXXXXXXXXXXXXXXXX
VEEV INH9813-K3E GGCGCGCCTAATACGACTCACTATAG                                                                              13626
9813-Sequenced                                                                                                         11450
```
