# Supplementary material for: Long-term persistence of viral RNA and inflammation in the CNS of macaques exposed to aerosolized Venezuelan equine encephalitis virus
Source: PLoS Pathog. 2022 Jun 13;18(6):e1009946. doi: 10.1371/journal.ppat.1009946 (PMC9232170; doi:10.1371/journal.ppat.1009946)
Supplement: Attachment — Submitted filename: VEEV PLoS Pathogens response to reviewers.docx [file ppat.1009946.s004.docx]

Reviewer's Responses to Questions

Part I - Summary

Please use this section to discuss strengths/weaknesses of study, novelty/significance, general execution and scholarship.

Reviewer #1: The manuscript describes the long-term persistence of vRNA and inflammation in the CNS of NHPs exposed to VEEV via the aerosol route of exposure. The challenge virus was VEEV INH-9813 that was generated via reverse genetics and was delivered via small particle aerosol. The paper addresses some key questions regarding the NHP model of VEEV, particularly regarding the more long-term sequelae associated with disease. Overall, the study is well done, however, it would benefit from including more experimental and analysis details that will enable future comparisons possible.

Reviewer #2: In this manuscript, the authors present their finding on small aerosol exposure of Cynomolgus macaques with Venezuelan equine encephalitis virus. While previous studies have been published on aerosol exposure of primates with VEEV, this study utilized clonally derived stocks of the INH-9813 strain selected for preclinical efficacy studies under the FDA animal rule. Additionally, this study employed radiotelemetry to track fever and measure electroencephalography and intracranial pressure providing greater insight into CNS disease caused by VEEV infection. Results from this analysis provided additional markers that could be used to assess drug/vaccine efficacy. Lastly, the authors report that VEEV viral RNA along with pro-inflammatory cytokines/chemokines and leukocytes are detectable in CNS tissues associated with pathological changes at 50 or more days post infection. This finding is of significant importance for chronic VEEV disease. Aside from some minor grammatical errors and omission of detail on the control animals the manuscript is well written and discussed; however, it is this reviewers opinion that the manuscript would benefit from an assessment of the impact of gender/age on the results even if minor or inconclusive.

Reviewer #3: This manuscript offers new insights into VEEV infection in nonhuman primates exposed to small particle aerosols. The strengths of this study lie in using an infectious clone of VEEV and including telemetry data to track neurological abnormalities that are sometimes absent by cage-side observation alone or traditional data acquisition such as blood work and virologic analyses. However, unfortunately the manuscript lacks detail and could be executed in a better way with regard to attention to detail, writing, material references and discussing the findings within a broader and more relevant context.

________________________________________

Part II – Major Issues: Key Experiments Required for Acceptance

Please use this section to detail the key new experiments or modifications of existing experiments that should be absolutely required to validate study conclusions.

Generally, there should be no more than 3 such required experiments or major modifications for a "Major Revision" recommendation. If more than 3 experiments are necessary to validate the study conclusions, then you are encouraged to recommend "Reject".

Reviewer #1: 1. Key details regarding methodology are lacking. Please review the Materials & Methods section and ensure that sufficient details are provided. Some specific examples that should be addressed: the section on cells/media are missing; details regarding the aerosol exposure such as maximal exposure time, sampling efficiency of the AGI for VEEV, location of sampling for determination of the exposure dose (methods say chamber, but is it at the point of the head only device?). For EEG/ICP/Temp: how long was data acquisition stopped and was this the same time every day?

Cells & media section has been added.

These studies were being performed as we made the transition to the volume-based aerosols, so the original methods included both. A review of the NHP data published in this study indicated however that all of the macaques were exposed for 10 minutes, so the methods section on aerosol exposures was revised accordingly. In addition, we added a statement to the aerosol exposure section that the AGI is attached to the head-only chamber in the area where the NHP is breathing (the ‘breathing zone’). Sampling efficiency was not empirically determined in this study as it is not relevant to the inhaled dose; in prior studies the sampling efficiency for the AGI we use is approximately 97%. Text was added for the EEG/ICP/Temperature acquisition section regarding when telemetry was stopped (just after morning observations) and duration (maximum of 15 minutes).

2. For clinical scoring the authors indicate that they increased observations when NHPs reached severe disease (score of 10) and monitored animals every 8 hrs, including overnight. Given that they were also monitoring circadian patterns, how did they account for the overnight disturbances in the circadian pattern data when observations were increased as this would likely create noise in the data.

VEEV only rarely causes lethal disease in macaques. None of the macaques in these studies reached a score that required overnight observations. We have amended the manuscript to indicate this, our scoring system is one that we use for all encephalitic viruses including viruses like Rift Valley Fever virus and eastern equine encephalitis virus.

3. The method section for pathology requires more detail. How long were samples fixed in formalin prior to removal from biocontainment? Provide a reference or detail for tissue/slide preparation. Were samples scored by a boarded pathologist?

Dr. Pandrea, a co-author on this paper, is a boarded pathologist with considerable experience in evaluating viral pathologies in nonhuman primates. Tissues were embedded in paraffin and sections cut and placed on slides by the Histology Lab in the McGowan Center for Regenerative Medicine at the University of Pittsburgh. We have provided the information they sent us on how tissues are embedded and slides cut.

4. The pathology section needs more detail to be convincing. The main conclusion based on path results is that there is evidence of diffuse inflammation throughout CNS regions after recovery from physiological responses. Based on the data, it appears that the majority of animals had mild to no lesions (Fig 13G). The H&E images appear to be focal lesions, but due to the increased magnification of the panels from left to right, appear more diffuse. The other important question that was not addressed was whether the areas of inflammation observed by histopath correspond to either vRNA or local cytokine production. Immunostaining would be far more convincing here.

Changed the description in the results so it now reads “evidence of mild, diffuse inflammation throughout CNS regions of some VEEV-infected macaques (Fig 13G),…”

With regards to whether the areas of inflammation correspond with vRNA or inflammatory cytokines, this is an area that is the subject of a separate large-scale investigation and we feel inclusion of such information is beyond the scope of this manuscript and would be better suited to a separate submission at a future date.

5. In looking at Fig 1, it is difficult to appreciate a biphasic fever. The low dose group is perhaps the most convincing (with the exception of NHP 6-19). In general, the fever response appears more undulating with multiple peaks and valleys. Can the analysis for delineating the fever phases be described in more detail with respect to the statistical analysis that was used to determine if data points represented a unique phase. What is the definition of fever in the model (x degrees above baseline)?

We have added information to the Temperature data analysis in the Methods section to explain better how fever is calculated in our model. Each animal has baseline data which is used to calculate temperature deviations from baseline for that individual animal. There is a biphasic fever seen in every animal but the slight variations in onset and maximum fever make it difficult to discern when multiple animals are shown in one graph as seen in figure 1. Added the word “generally” to describe biphasic fevers.

6. Could the 'noise' in Fever 2 be due to it being more than a single phase and actually multiple fevers during that time?

We have added a statement to that effect. The period 2.5-8 dpi encompasses the second febrile period for all of the macaques in this study as well as in prior studies.

7. Figure 5: Since the significant difference in circadian index is seen in F2, could this be due to the fact that you increased observations during that time period and disturbed the natural sleep cycle of the animals (ie: is this artificially induced)?
As mentioned in the response to question 2 above, there were no overnight observations of these animals.

8. what is the background in Fig 8 A & C that is subtracted? It doesn't make sense to have such low titers by PCR relative to the infectious titers shown.

Non-specific amplification of vRNA was observed in the 0hr time-points, therefore the “background” referred to in the paper is the 0hr time-points subtracted from each of the following time-points for each corresponding animal/sample. By subtracting the non-specific background, we were more stringent in our analyses of long-term presence of vRNA.

9. The sequence of the infectious clone virus stock should be provided particularly since the authors correctly point out that prior studies used cell culture passaged virus that was not genotyped. This paper should indicate in more detail how the virus was rescued and number of passages that were required to obtain the stock that was characterized and used for aerosol challenge.

The cDNA clone sequence is in a separate manuscript currently under review: Chengqun Sun, Christina L. Gardner, Matthew D. Dunn, Derek W. Trobaugh, Douglas S. Reed, Amy L. Hartman and William B. Klimstra (2021). In vitro and in vivo phenotypes of Venezuelan, eastern and western equine encephalitis viruses derived from cDNA clones of human isolates. *Under review at PLOS NTD.* Viruses were generated by electroporation of in vitro synthesized genomic RNA into BHK cells followed by harvesting at ~24 hours post electroporation, clarification of supernatants and reinfection of Vero cells at an MOI of 5-10. After ~24 hours, Vero cell supernatants were clarified and partially purified by discontinuous sucrose gradient sedimentation (20%/60%) and pelleting through 20% sucrose followed by resuspension in HEPES buffered OPTIMEM medium. Virulence of the stocks was confirmed by aerosol LD_50_ analysis in mice. These methods are briefly reported here and a reference is given to a manuscript with more detailed methods.

10. The authors mention the significance of a biphasic fever in human cases of VEEV, however, in recent reviews it was shown that the observation of biphasic fever was actually not a common occurrence. Can the authors comment on this? Reference = PMID 30282570

While the article mentioned is of interest because it reports on lab-acquired infection, we do not find that it supports the statement that biphasic fever is not a common occurrence. Biphasic fever is only discussed in two places in that article. The first is in reference to aerosol exposure of laboratory personnel, whose vaccination status is not made clear. Vaccination with TC-83 or C-84 could alter the disease course. The poor protection conferred by these vaccines against aerosol challenge, particularly C-84, is well-recognized. Nor is it clear to what strain/subtype of VEE these individuals were exposed. TC-83 and C-84 do not protect well against enzootic strains, which is another reason neither has moved forward as a VEE vaccine. Aerosol exposure of macaques to Mucambo, which was previously a IIIA subtype of VEE but is no longer classified as VEE, can cause a monophasic fever (Reed et al, 2005 J. Inf. Dis). The second location in the manuscript cited by the reviewer is in reference to individuals who received the live, attenuated TC-83 vaccine. This is unlikely to apply to virulent VEE. Our review of the literature concerning natural outbreaks of epizootic VEE lists biphasic fever as a commonly seen symptom.

11. A tremendous amount of work and data are shown with respect to inflammatory mediators. The discussion could be expanded further to put these data in context with the other data that are presented.

We have expanded the discussion to include the inflammatory mediators as requested, as well the traumatic brain injury gene expression.

Reviewer #2: None.

Reviewer #3: Though no additional experiments are suggested, careful and extensive re-writing of the text and re-framing of the findings are required. Please see extent of comments in next section.

________________________________________

Part III – Minor Issues: Editorial and Data Presentation Modifications

Please use this section for editorial suggestions as well as relatively minor modifications of existing data that would enhance clarity.

Reviewer #1: 1. Figure 13: Text refers to 13G, however, the chart in Fig 13 is not labelled

2. There are several broad/general comments (particularly in the introduction) that require clarification and references. Specifically, the first sentences in paragraphs #1 and #2 as they imply that aerosol transmission is a typical route of transmission (para 1) and is highly infectious in humans via aerosol (para 2). I suspect the authors are referring to animal studies that have shown this to be the case.

We amended paragraph 1 in the introduction to read “naturally mosquito-transmitted but laboratory accidents have shown it can also cause disease when inhaled” to make sure it is clear that it is not a natural or typical route of infection but which has happened in humans. We have also re-worded the first sentence in the second paragraph which was somewhat redundant.

3. Under challenge virus in M&M, the last sentence refers to "dose-step infection of CD-1 mice". The reviewer assumes this was an LD50 study. What is the "typical virulence" the authors refer to? Is this the known LD50 in mice? Please be more specific.

Removed the word ‘typical’ and added more specific language.

Reviewer #2: The purpose of the study in the last paragraph of the introduction could be written better for clarity and EEG and ICP should be written out at first introduction.

Rewritten as requested, and EEG and ICP are written out as requested.

Details on the mock/uninfected animals should be included in the methods.

Details on mock-infected macaques were added to Aerosol Exposure section in Methods. Since all ‘uninfected’ were in fact mock-infected, we have replaced “uninfected” with mock-infected throughout the manuscript.

SIV, STLV and SRV should be spelled out under general animal procedures.

Done

Aerosol Exposure "attached to the chamber and operated at...

It is not clear what the reviewer is requesting here. We have amended that section in accordance with reviewer #1 so it now states “To determine inhaled dose, an all-glass impinger (AGI; Ace Glass, Vineland, NJ) was attached to the head-only chamber and operated at 6 lpm, -6 to -15 psi. The AGI is attached in the breathing zone of the animal.”

EEG data analysis - be consistent in how the HZ is presented and the use of parenthesis

Corrected.

Brain cell isolation = centrifuged at 500 x gravity

While we have changed this as the reviewer requested, x g is commonly accepted in the scientific community to refer to gravity when used in conjunction with centrifugation.

RNA isolation = phase was collected then combined

Corrected

In the results section the Johnston Ref should be detailed in the first paragraph and it's worthwhile to state that regardless of the dose that exposure to aerosolized VEEV was non-lethal during the period examined.

Done

Need to be consistent with use of average vs median delta T in the second paragraph of the fever response.

Should have been median, not average. That has been corrected.

Provide a description of mock infected animals in results and be consistent when referring to mock infected vs. uninfected. These were the same? Was the temperature of mock infected animals observed? Was telemetry done on those animals as well or does this data come from a previous study.

Mock-infected and uninfected were the same. We have corrected manuscript accordingly to say only mock-infected. Telemetry was done on these animals and was used for comparison purposes as described in the manuscript.

Were there any gender or age differences in the results? This is important for NIAID/FDA.

Given the number of animals exposed, and that part of the focus was on dose, it is difficult to elucidate statistically significant differences based on sex or age. At this point, the data do not indicate a difference based on age. Female macaques did have lower febrile responses but there are differences in age, weight, and dose that confound the interpretation of this data. At this point it is merely suggestive. We have added a statement to the second paragraph in the Results section that covers this.

Second paragraph of discussion TrD, which was originally isolated ...

Not sure what the reviewer is requesting be changed.

Discuss gender/age differences even if nothing significant was observed

Added to Results, second paragraph. Also added to second paragraph of Discussion.

Most of the figures compare mock vs VEEV in that order with the exception of Figure 11. Suggesting making this consistent throughout.

Corrected.

Reviewer #3: General:

o Remove double spaces throughout manuscript

We have corrected all that we could find. If any were missed, please let us know.

o Replace “infection” with “exposure”

We believe this is an individual choice and saying infection rather than exposure accurately reflects what we are reporting, that macaques were infected with the aerosol route with VEEV.

Abstract:

o Spell out infectious clone (IC)

As stated in the abstract, IC is the subtype of VEEV used, not that we used an infectious clone. Earlier in the same sentence we use the words infectious clone.

o Human infection should be replaced with human case of VEEV

o Replace CNS with CSF or CNS tissues

Spelled out CNS. Since it was found in both, more appropriate to be inclusive.

o Replace “viral” with “VEEV” genomic nucleic acid
Done

o Rephrase “persistence of virus replication” with “persisting virus” unless authors have evidence of active replication such as isolation of infectious VEEV

as stated in abstract “These data are consistent with persistence of virus replication and/or genomic RNA”

Introduction

o Consider splitting up the first sentence or leave out “cause disease when inhaled” and explain in subsequent paragraph that the inhalational route is of relevance with regard to biothreat purposes.

Initial sentence has been reworded based on reviewer #1. First sentence in second paragraph of introduction indicates the potential biothreat concern.

o Replace “instrumental” with “important study” or another more suitable statement

Done

o Explain why crab-eating macaques are useful, or relevant models of alphavirus infection. Why was not a mouse model used for the study, especially as wildtype VEEV is more lethal pin mice than in nonhuman primates as the authors state?

As stated in the Introduction (italicized here to emphasize why cynomolgus macaques are useful as a model): “Wild-type VEEV is generally lethal in mice, *but in both rhesus and cynomolgus macaques, VEEV disease is typically self-limiting and the animals eventually recover, much like the majority of infected humans*”

o Spell out “FDA”

Done

o What do the authors consider as “low passage” virus stock? Are genetic changes acquired during cell culture passage of virus, and if so do they contribute to phenotypic changes as well (for example attenuation to in vivo)?

The viruses used in the studies are generated from a cDNA clone with initial electroporation supernatants passaged once at high multiplicity in Vero cells. In mouse aerosol LD_50_ assays (and is all other measures of virulence), the initial electroporation supernatant and the Vero passaged material are phenotypically indistinguishable. The passage is done such that high aerosol doses can be generated.

o Spell out “NHP”

Done

o In the last paragraph the authors state that disease in NHPs has only been characterized observationally, but then first objective of this study was to further characterize disease which is a contradiction

We have re-written the last paragraph to indicate that it is only the neurological component of the disease that has been characterized observationally and that one of the goals of this work was to use telemetry to better define neurological disease in the macaques.

Material and Methods

o Please add the ALAAC and PHS accreditation and approval numbers specific to your institution

Added.

o Challenge virus: Was one or multiple strains used? If exposure was only performed with one infectious clone, then please state accordingly

as indicated in the text, macaques were challenged with viruses derived from infectious clones of either INH-9813 or Trinidad Donkey (TrD); only two macaques were challenged with TrD

o Have the authors confirmed the sequence of the infectious clone after cell culture passage and prior to confirmation of virulence in mice to ensure that no additional mutations were acquired through propagation in Vero cells?

In addition to the validation of phenotype of stocks generated from the clones described above and in the manuscript, we have compared the sequences in the cDNA clones for deviation from VEEV consensus sequences and type specific changes versus consensus sequences of IA/B (TrD) and IC (INH98-13) strains available in Genbank. This evaluation is a component of a manuscript submitted separately and under review. We have not sequenced the Vero cell amplified material but we have shown that it is identical in mouse aerosol LD_50_ to material derived from the initial BHK cell electroporation supernatant, which is highly indicative of limited effects of artifactual heparan sulfate binding. If artifactual HS binding mutations affect virulence with after a single high efficiency electroporation of BHK cells, we are uncertain as to how this could be addressed in an effort to generate high titer aerosol infection doses.

o Correct 37C to 37 °C

Corrected

o What was the cut-off for the PRNT, was it 50% or 80%? Also, please add material references for the commercial mouse antibody

The text in the methods section on PRNT was copied and adapted from another manuscript. Since we are not showing any PRNT data in this manuscript, we have removed that section from the methods.

o Add a space between “200” and “mg/kg”

Done

o What is “Beuthanasia”? Is it pentobarbital? Please reference any commercial product used and add the dosage.

Beuthanasia is the commercial name of sodium pentobarbital; we have corrected the manuscript to say sodium pentobarbital and the vendor. Dosage was already listed at 200 mg/kg.

o Was euthanasia or death confirmed? How was death confirmed?

Yes, it was confirmed. We have added text to indicate it was confirmed by cessation of heart beat.

o Change “tracheal tube” to “endotracheal tube”

Done

o How long were the aerosol exposures? Was this a time-calculated run or accumulated dose based on respiration?

Exposures were 10 minutes in duration. These experiments took place as we were transitioning to volume-based aerosols but all of the VEEV aerosols described in this study were duration, not volume based. We have updated the text to reflect this.

o What is lpm? Do the authors mean 16 liters per minute? Please clarify

lpm is liters per minute. In our opinion, this is a commonly accepted/understood abbreviation

o What was the duration of particle size measurements? It was started at 5 minutes into the exposure and lasted until which minute? Was it the same across all sprays?

We have added text to indicate it was 30 seconds in duration. It was the same across all sprays.

o How was baseline behavior of nonhuman primates normalized to behavior recorded post-exposure?

We’re not sure what the reviewer is asking. Baseline behavior, that assessed by observation, was not normalized to post-exposure behavior. Behavior was scored according to the criteria laid out in the scoring section of the methods.

o The clinical scoring example in parenthesis is not necessary, please remove. Is it clear that a cumulative score of 10 triggered euthanasia, but please indicate if secondary parameters were used to meet the euthanasia criterion (such as blood parameters like glucose or leucocytes, or extreme hypothermia).

Considering that other reviewers felt the score was important, with which we would agree, we would prefer to leave it in as it is helpful for the reader to understand how the observations were done. No animals in this study became moribund or met criteria for early euthanasia; no secondary parameters were used or would have been used for euthanasia decisions. Fatal infection from VEEV in macaques is quite rare. We have seen that clinical parameters such as granulocyte counts could be useful as an early endpoint in EEEV infection of macaques. We do see lymphopenia in VEEV infection but this is indicative of disease, not death.

o How was plasma obtained? Please add blood tubes, centrifugation details etc.

Blood tube was already listed (EDTA). Centrifugation details added.

o Please list all tissues collected during necropsy including the ones from different regions of the brain, was the collection different between nonhuman primates? Were different tissues collected from controls?

Tissues collected from the CNS are shown in Figure 9. There were no differences in how tissues were collected between nonhuman primates or between VEEV-infected and mock-infected controls.

o The nucleic acid isolation and PCR sections are missing material references.

Added.

o Please remove “in-house”

Removed

o How many 10-fold dilutions were used to generate a standard curve for quantification of gene copies by RT-PCR?

5-6 10-fold serial dilutions were created and included in the qPCR

Results

o Are the delivered dose ranges that the authors used for grouping of the exposed nonhuman primates biologically relevant?

The doses reported here are within the ranges used in prior studies infecting cynomolgus macaques with VEEV to study pathogenesis of VEEV after aerosol exposure or to evaluate potential vaccines.

o How did the authors determine fever? What were the core temperatures measured during the febrile periods?

We have added more detail to the Temperature data analysis section of the Methods to explain how fever is determined. We used residual temperatures (actual minus predicted) to determine fever. Core body temperatures were only recorded in macaques that used implants for electrocardiography. For temperature only or electroencephalography, the implants are subcutaneously. In our experience, covering 21 years and a considerable number of macaques, while subcutaneous temperatures are lower than core, the pattern of temperature changes from diurnal variation, fever, or hypothermia are the same whether measured from the core or subcutaneous.

o How were significant differences in clinical signs between the exposed groups assessed?

Significant differences were seen between post-infection and pre-infection (baseline) observations, but not between exposed groups. Significance was determined by one-way ANOVA analysis with multiple comparisons, as described in Fig. 3.

o What does it mean if there is “aggregate decrease in all wavebands”?

Removed the word aggregate so it is clearer that there is a significant decrease in all wavebands.

o Please rephrase: “As has been reported previously with VEEV infection of non-human primates (5, 6), after aerosol exposure to INH-9813, there was a significant drop in white blood cell counts of macaques in the first five days after infection, and most of that loss was in the lymphocyte population.

Done

o Please use more accurate terms within the hematology section and include results for glucose.

We are unsure what the reviewer means by “more accurate terms”. The terms we have used are those reported by our analyzers and are commonly used in reporting hematology results. Glucose was not shown in our paper because the results did not show any substantial change over the course of the disease (see graph). While we are aware that VEEV has been found in the pancreas of rodent species, this has not been seen in nonhuman primates or humans to our knowledge. A literature search failed to turn up any references to VEEV affecting glucose levels in primates.

o Viremia section: This section lacks detail, especially to the level of virus detected, please add how much virus was detected (titer and VEEV copies/mL)

We have added detail to this section as requested.

o Were increases in cytokines significantly higher in nonhuman primates with higher viral load and if so, what is the level of significance?

A comparison of viral load in the CNS tissues (Fig 9) and cytokine/chemokine levels shown in Fig 10 did not show an association between viral load and cytokine levels.

o Please explain what presence of the three markers of traumatic brain injury suggests

The text currently states:” This may suggest there is some disruption of the blood-brain barrier on day 6 after infection with VEEV but it is not as severe as that seen with EEEV.”

Discussion

o How specifically are the telemetry data useful and informative for future evaluation of countermeasures. What if use of telemetry and implantation are not possible? Is there another surrogate biomarker that could bridge this gap?

The data here show the similarity between the disease in nonhuman primates and what has been reported in humans. Telemetry has been used in multiple instances to demonstrate vaccine efficacy in nonhuman primates, including 6 studies with VEEV. Considering the cost of nonhuman primates, the additional cost of telemetry implants (especially those that only measure temperature) is not substantial and the value gained far outweighs the cost. Particularly for diseases like VEEV where the outcome is not a reduction in mortality but a reduction in morbidity. We have expanded the discussion on EEG and ICP telemetry.

o Please explain what low- and high-passage virus stock means in the context of virulence studies. What are changes of concern? Was the infectious clone sequenced in the current study?

The sequence of the TrD clone has been published in the reference listed in the methods and the INH9813 clone is in a manuscript currently under review. The acquisition of positively charged residues in the E2 attachment glycoprotein that increase virion interaction with heparan sulfate are typically the first type of cell-adaptive mutations that arise during alphavirus passage in cell culture (at least that can be readily identified phenotypically). Virus derived from the both the TrD and INH9813 cDNA clones as described in Materials and Methods has been evaluated for this phenotype and found to have minimal HS binding. These results are a component of a separate manuscript that is currently under review. The INH9813 cDNA clone was generated by us from a low-passage stock that we identified as possessing a single mutation that conferred enhanced HS binding and decreased virulence in mice. This mutation was eliminated from the cDNA clone by reversion to a VEEV IC type-specific consensus reside at that position.

o Please list and discuss limitations of your study.

The discussion includes limitations of the telemetry used in the study as a trigger for treatment, the failure to find live virus in the nostrils of the VEEV-infected macaques and that the persistence of viral RNA in the CNS as detected by q-RT-PCR needs to be further explored and verified as indicative of intact, replicating virus.

o Please discuss implications and benefit of your findings towards the improvement of future studies.

The discussion includes sections regarding the utility of telemetry in determining efficacy, a new section on the inflammatory response in the CNS, and in the final paragraph highlights that the persistence of viral RNA in the CNS, if confirmed to be intact virus, would be a useful marker in evaluating vaccine or therapeutic efficacy in future studies.
