# Supplementary material for: Long-term persistence of viral RNA and inflammation in the CNS of macaques exposed to aerosolized Venezuelan equine encephalitis virus
Source: PLoS Pathog. 2022 Jun 13;18(6):e1009946. doi: 10.1371/journal.ppat.1009946 (PMC9232170; doi:10.1371/journal.ppat.1009946)
Supplement: Attachment — Submitted filename: response to reviewers.docx [file ppat.1009946.s005.docx]

Reviewer #1: 1. The authors responded to comment 1 by stating, "Sampling efficiency was not empirically determined in this study as it is not relevant to the inhaled dose; in prior studies the sampling efficiency for the AGI we use is approximately 97%." The reviewer disagrees that this is not relevant as it will ultimately influence the amount of virus that can be recovered and estimate of the dose. Given that the aim was to use a well characterized stock and further characterize the model, this is a major point that should be empirically tested. Recovery efficiencies will vary by agent and can contribute to differences observed in animal models if it is not well characterized prior to challenge.

We went back to our original data and from that calculated what we believe to be the sampling efficiency. We did this by calculating the ‘hypothetical’ aerosol concentration (comparing pre- and post-aerosol nebulizer contents, which assumes 100% of the difference in total virus between the two was virus that was aerosolized) and from that calculating the hypothetical impinger concentration assuming 100% capture in the liquid in the impinger. That was compared with the actual impinger concentration. This came out to an average of 98% (i.e., the aerosol concentration calculated by the actual impinger contents was within 2% of the hypothetical aerosol concentration) although there was run-to-run variability. This fits well with the sampling efficiency we have seen with other viral agents and bacteria, although there is variability between agents and run-to-run/day-to-day variability based on methods used for quantifying the agent. We would reiterate again that this efficiency calculation does not change how we calculate the inhaled dose which is the product of the accumulated tidal volume and virus concentration in the aerosol, which is determined from what is collected in the impinger [1-4]. This calculation has been used for decades, including the calculation of inhaled doses in nonhuman primates exposed to VEEV over the last 20 years by the senior author on this manuscript and by others [5-13]. If this is extended more broadly to other experimental respiratory infections caused by other viruses and bacteria, the number of references using this methodology, including the same type of impinger, flow rates, and sampling methods, is considerable. We do agree that there are differences based on the choice of sampling device used, and that this could change the apparent aerosol concentration and thereby the calculated inhaled dose. A more thorough analysis of the choice of sampling devices, chambers, and nebulizers used for aerosolization of VEEV to optimize aerosol delivery and calculations of inhaled dose would be of value to the aerobiology community but is beyond the scope of this paper, which is about the disease caused by inhalation of VEEV in the nonhuman primate model. For this reason, we intentionally used the same type of all-glass impinger that has been used for the last 2+ decades on all nonhuman primate studies conducted with aerosolized VEEV at the University of Pittsburgh, as well as USAMRIID, UTMB, and Tulane, as this gives us comparability with those results.

1.	Reed DS, Xhillari D, Weiss AL, Jaeger RJ. CHAPTER 13 Aerosol Exposure to Pathogenic Bacteria and Virus Particles: Standard Operating Procedure. Aerobiology: The Toxicology of Airborne Pathogens and Toxins: The Royal Society of Chemistry; 2016. p. 445-59.

2.	Roy CJ, Pitt, M.L.M. Infectious Disease Aerobiology: Aerosol Challenge Methods. In: Swearengen JR, editor. Biodefense Research Methodology and Animal Models. Second ed. Boca Raton, FL: CRC Press; 2012. p. 65-80.

3.	Roy CJ, Pitt, L.M. Infectious Disease Aerobiology: Aerosol Challenge Methods. In: Swearengen JR, editor. Biodefense: Research Methodology and Animal Models. Boca Raton, FL: CRC Press; 2005. p. 61-76.

4.	Hartings JM, Roy CJ. The automated bioaerosol exposure system: preclinical platform development and a respiratory dosimetry application with nonhuman primates. J Pharmacol Toxicol Methods. 2004;49(1):39-55. PubMed PMID: 14670693.

5.	Ma H, Lundy JD, Cottle EL, O'Malley KJ, Trichel AM, Klimstra WB, et al. Applications of minimally invasive multimodal telemetry for continuous monitoring of brain function and intracranial pressure in macaques with acute viral encephalitis. PLoS One. 2020;15(6):e0232381. Epub 2020/06/26. doi: 10.1371/journal.pone.0232381. PubMed PMID: 32584818.

6.	Ma H, Lundy JD, O'Malley KJ, Klimstra WB, Hartman AL, Reed DS. Electrocardiography Abnormalities in Macaques after Infection with Encephalitic Alphaviruses. Pathogens. 2019;8(4). Epub 2019/11/21. doi: 10.3390/pathogens8040240. PubMed PMID: 31744158.

7.	Reed DS, Glass PJ, Bakken RR, Barth JF, Lind CM, da Silva L, et al. Combined alphavirus replicon particle vaccine induces durable and cross-protective immune responses against equine encephalitis viruses. J Virol. 2014;88(20):12077-86. Epub 2014/08/15. doi: 10.1128/JVI.01406-14. PubMed PMID: 25122801; PubMed Central PMCID: PMC4178741.

8.	Dupuy LC, Richards MJ, Reed DS, Schmaljohn CS. Immunogenicity and protective efficacy of a DNA vaccine against Venezuelan equine encephalitis virus aerosol challenge in nonhuman primates. Vaccine. 2010;28(46):7345-50. Epub 2010/09/21. doi: 10.1016/j.vaccine.2010.09.005. PubMed PMID: 20851089.

9.	Koterski J, Twenhafel N, Porter A, Reed DS, Martino-Catt S, Sobral B, et al. Gene expression profiling of nonhuman primates exposed to aerosolized Venezuelan equine encephalitis virus. FEMS Immunol Med Microbiol. 2007;51(3):462-72. Epub 2007/09/27. doi: FIM319 [pii]

10.1111/j.1574-695X.2007.00319.x. PubMed PMID: 17894805.

10.	Reed DS, Lind CM, Lackemeyer MG, Sullivan LJ, Pratt WD, Parker MD. Genetically engineered, live, attenuated vaccines protect nonhuman primates against aerosol challenge with a virulent IE strain of Venezuelan equine encephalitis virus. Vaccine. 2005;23(24):3139-47. PubMed PMID: 15837213.

11.	Reed DS, Lind CM, Sullivan LJ, Pratt WD, Parker MD. Aerosol infection of cynomolgus macaques with enzootic strains of Venezuelan equine encephalitis viruses. J Infect Dis. 2004;189(6):1013-7. PubMed PMID: 14999604.

12.	Ko SY, Akahata W, Yang ES, Kong WP, Burke CW, Honnold SP, et al. A virus-like particle vaccine prevents equine encephalitis virus infection in nonhuman primates. Sci Transl Med. 2019;11(492). Epub 2019/05/17. doi: 10.1126/scitranslmed.aav3113. PubMed PMID: 31092692.

13.	Pratt WD, Gibbs P, Pitt ML, Schmaljohn AL. Use of telemetry to assess vaccine-induced protection against parenteral and aerosol infections of Venezuelan equine encephalitis virus in non-human primates. Vaccine. 1998;16(9-10):1056-64.

Reviewer #1: 2. The fact that the viral stock that was used in the challenge was not well characterized remains a major concern. This material should be fully sequenced and characterized. Even a single passage in cell culture can result in SNPs that may or may not influence pathogenesis. While confirmation of virulence in the mouse model does alleviate some concern, it doesn't necessarily suffice for proper stock characterization. Given that the authors point out the importance of this, it is difficult to justify publication without this.

Reviewer #3: Given the potential impact of single nucleotide mutations on the phenotype of the viral stock used, and the author's discussion of low versus high passage stocks used for aerosol exposure, it is important to provide confirmation that the Vero cell passaged stock used for the NHP exposure matches the reference sequence. A phenotypic confirmation in mice or in vitro is not sufficient. Please provide confirmation of the sequence in your response.

Our infectious clone of INH-9813 was sequenced by Sanger sequencing of overlapping RT-PCR amplicons. No differences with the cDNA clone sequence were observed. Coverage did not include the 5’ and 3’ 20 nucleotides of the stock virus reflecting primer binding sites. This has been added in the methods section; the primers used and the results of the sequencing will be included in supplemental information.
